# Change in physical activity and quality of life in endometrial cancer survivors receiving a physical activity intervention

**DOI:** 10.1186/s12955-019-1154-5

**Published:** 2019-05-27

**Authors:** Michael C. Robertson, Elizabeth J. Lyons, Jaejoon Song, Matthew Cox-Martin, Yisheng Li, Charles E. Green, Bernardine M. Pinto, Cindy L. Carmack, Carol Harrison, George Baum, Karen M. Basen-Engquist

**Affiliations:** 1 Center for Energy Balance, Department of Behavioral Science, MD Anderson Cancer Center, Cancer Prevention Building, Unit 1330, 1155 Pressler St, Houston, TX 77030 USA; 2 Health Promotion & Behavioral Sciences, University of Texas School of Public Health, 1200 Pressler Street, Houston, TX 77030 USA; 3 Department of Nutrition and Metabolism, School of Health Professions, University of Texas Medical Branch at Galveston, 301 University Boulevard, Galveston, TX 77555-1124 USA; 4 Department of Biostatistics, The University of Texas MD Anderson Cancer Center, Pickens Academic Tower, 1400 Pressler St, Houston, TX 77030 USA; 5 Department of Pediatrics, McGovern Medical School, The University of Texas Health Science Center, 6431 Fannin St, Houston, TX 77030 USA; 6 College of Nursing, University of South Carolina, 1601 Greene Street, Room 302B, Columbia, SC 29208-4001 USA; 7 Department of Palliative Care, Rehabilitation, and Integrative Medicine, MD Anderson Cancer Center, Cancer Prevention Building, Unit 1330, 1155 Pressler St, Houston, TX 77030 USA; 8 Adult and Child Consortium for Health Outcomes Research and Delivery Science, University of Colorado at Denver-Anschutz Medical Campus, Aurora, CO USA

**Keywords:** Endometrial cancer, Physical activity, Quality of life, Cancer survivors

## Abstract

**Background:**

Endometrial cancer survivors are at an increased risk of poor quality of life outcomes. Physical activity is positively associated with general quality of life in this population, however, little is known about how changes in physical activity may be associated with changes in specific aspects of quality of life. The aim of this secondary data analysis was to explore the relationships between change in physical activity and change in physical, mental, social, and other aspects of quality of life in endometrial cancer survivors receiving a physical activity intervention.

**Methods:**

Endometrial cancer survivors (*N* = 100) participated in a telephone-based physical activity intervention for six months. At baseline and post-intervention we measured physical activity via accelerometry and ecological momentary assessment, and quality of life via the Short Form Health Survey (SF-36), the Quality of Life of Adult Cancer Survivors instrument, the Brief Symptom Inventory, the Pittsburgh Sleep Quality Index, and the Perceived Stress Scale. We conducted structural equation modeling path analyses to investigate how physical activity post-intervention was associated with the quality of life measures’ subscales post-intervention, adjusting for baseline levels and potentially confounding covariates.

**Results:**

Increasing physical activity was positively associated with improvements in general health (*p* = .044), role limitation due to physical health (*p* = .005), pain (*p* = .041), and somatic distress (*p* = .023). There was no evidence to indicate that change in physical activity was associated with change in other aspects of quality of life.

**Conclusions:**

Endometrial cancer survivors are at higher risk for suffering from challenges to physical quality of life, and findings from this study suggest that increasing physical activity may alleviate some of these problems. Further research is needed to determine whether other aspects of quality of life are linked to change in physical activity.

**Trial registration:**

Trial registration number: NCT00501761

Name of registry: clinicaltrials.gov

Date of registration: July 16, 2007.

Date of enrollment: June 16, 2005.

## Introduction

Endometrial cancer is the fourth most common cancer among U.S. women, and its high five-year survival rate has contributed to a large and growing population of survivors [1]. While its link with endometrial cancer recurrence is not well studied, physical activity is associated with reduced endometrial cancer incidence [2] and reduced five-year all-cause mortality among survivors [3]. Endometrial cancer survivors suffer from high rates of obesity- and physical activity-related co-morbidities (e.g., type 2 diabetes) that are related to cancer-specific and overall mortality [4–7]. This population also faces marked challenges to quality of life outcomes, which are often linked to high rates of overweight and obesity and low adherence to health-protective lifestyle behaviors such as physical activity [8].

While evidence indicates that increasing physical activity can lead to improvements in quality of life in survivors of some types of cancer [9], there have been few studies that have investigated the nature of this relationship in endometrial cancer survivors specifically [10–12]. Furthermore, quality of life is a multi-dimensional construct, and limited literature has investigated which specific aspects of endometrial cancer survivors’ quality of life may be impacted by increasing physical activity. This population faces unique quality of life-related challenges, and a more comprehensive understanding of the potential benefits of increased physical activity is needed. The purpose of this secondary analysis was to investigate how change in physical activity over time related to change in multiple, specific measures of quality of life for endometrial cancer survivors receiving a physical activity intervention. We hypothesized that increased physical activity would be associated with positive changes in physical, mental, and general health-related quality of life outcomes.

## Methods

### Recruitment

Study methods are presented in greater detail elsewhere [13, 14]. Briefly, between January, 2007 and September, 2010 we recruited 100 women diagnosed with stage I, II, or IIIa endometrial cancer within the previous five years who had completed treatment, were cleared for exercise by their physician, and were not currently exercising regularly (i.e., not engaging in physical activity of moderate intensity on five or more days per week for 30 min or more, or vigorous intensity activity for 20 min or more at least 3 days per week, as measured by the Godin Leisure-Time Exercise Questionnaire [15]). We used a combination of both passive (e.g., sending letters of invitation) and active recruitment approaches (e.g., approaching potentially eligible participants at clinic visits).

We identified 643 endometrial cancer survivors potentially eligible for the study. Of those, 39 were ineligible on additional screening and 270 were incompletely screened (i.e., did not respond to letters and phone calls, and/or did not have appointments during the recruitment period). Of the remaining 334 women, 192 were not interested in the study and 42 were initially interested but did not complete either the consent process or the baseline assessment.

### Intervention and study design

The study design was a one-group, pre-post design. Each participant received a customized exercise prescription that was based on the results of baseline fitness tests. Each participant’s exercise prescription was determined by a professionally trained exercise physiologist who took into account subjective response to exercise and objective evaluations of heart rate, blood pressure, and VO2 max either measured directly or estimated during a graded exercise test. The exercise physiologist also took into account participants’ health status, medication use, risk factor profile, behavioral characteristics, personal goals, and exercise preferences in determining exercise prescriptions. We encouraged participants to engage in walking as their primary exercise modality and to gradually increase moderate-intensity walking over the course of the program with the goal of achieving 30 min of accumulated activity on most days of the week (i.e., a targeted exertion level corresponding to score of 12–16 on the Borg Rating of Perceived Exertion Scale; a rating of 15 corresponds to hard activity [16]). Due to evidence highlighting self-efficacy as an influential psychosocial correlate of exercise in endometrial cancer survivors, the current study’s behavioral intervention was based on Social Cognitive Theory; it was adapted from an intervention used by Pinto, Frierson, Rabin, Trunzo, & Marcus (2005) that was shown to be effective in breast cancer survivors [17]. The intervention, which has been described in greater detail elsewhere [13], was centered on the application of four methods of increasing self-efficacy: facilitating mastery experiences, verbal persuasion and feedback, vicarious experience and modeling, and improving affective states. Study staff provided 14 brief telephone counseling sessions during the 6-month intervention period, and provided supplementary content via mailed newsletters. Participants were called by phone counselors weekly for the first eight weeks. After that, the frequency of phone calls was gradually tapered, and each week participants were mailed progress reports and were instructed to re-visit their goals. We conducted assessments at baseline and at 6 months. At these time points we measured participants’ height and weight, quality of life, and physical activity for the five days before and after fitness assessments (with the exception that before baseline measurement we measured participants’ previous seven days of physical activity, not five).

### Measures

#### Physical activity

We assessed physical activity via electronic momentary assessment (EMA) and with participant-worn accelerometers. For EMA, we used Hewlett-Packard iPAQ RX1950 devices to have participants record their physical activity levels both (1) directly after daily exercise (real-time EMA), and (2) nightly. For accelerometry data, we used Actigraph GT1M accelerometers (Actigraph L.L.C., Pensacola, FL), which participants wore continuously for five days before and after each fitness assessment (seven days before baseline). As has been described in greater detail elsewhere [18], to minimize missing data we created a composite measure with these physical activity data. Based on the patterns of missing data, and to maximize the consistency of data across participants who engaged in different forms of physical activity (e.g., brisk walking, cycling, circuit training), this composite measure was based on a hierarchy in which participants’ real-time EMA data were used whenever possible; if this measure was missing for a particular day, then data for the composite measure were drawn from participants’ nightly EMA data. If neither of these measures were available, then that day’s accelerometer data were used to formulate the composite measure of physical activity.

#### Quality of life

Quality of life in cancer survivors is a complex, multifaceted construct that subsumes aspects of health, psychological well-being, physical and social functioning, and biological and physiological factors [19, 20]. As such, there is not necessarily a single “gold standard” for its measurement [21]. Furthermore, narrowly defining quality of life may leave researchers prone to overlooking clinically important outcomes. Accordingly, we conceptualized quality of life as being comprised as a constellation of factors that previous literature has suggested would be relevant in the context of oncology research. We assessed quality of life using: The 36-Item Short Form Survey (SF-36) [22], Quality of Life in Adult Cancer Survivors (QLACS) questionnaire [23], The Brief Symptom Inventory-18 (BSI) [24], the Pittsburgh Sleep Quality Index (PSQI) [25], and the Perceived Stress Scale (PSS) [26].

We administered the SF-36 because it is one of the most widely used and parsimonious instruments for measuring a wide range of aspects of quality of life. It consists of eight subscales to capture a core set of health-related quality of life outcomes, including: physical functioning (10 items), role limitations due to physical health (4 items), role limitations due to emotional problems (3 items), energy/fatigue (4 items), emotional well-being (5 items), social functioning (2 items), pain (2 items), and general health (5 items). Higher values indicate better quality of life. The SF-36 has demonstrated satisfactory psychometric properties [22], and in this study its internal consistency was high (Cronbach’s alpha (α) ≥ 0.80) for all subscales except for mental (α = 0.77) and general health (α = 0.77).

We included the QLACS questionnaire to provide additional insight into cancer survivor-specific aspects of quality of life (e.g., distress related to the prospect of cancer recurrence). QLACS was designed to measure various aspects of quality of life in long-term cancer survivors and to capture both generic and cancer survivor-specific domains of quality of life. Subscales include: negative feelings (4 items), positive feelings (4 items), cognitive problems (4 items), pain (4 items), sexual problems (4 items), energy/fatigue (4 items), social avoidance (4 items), financial problems (4 items), benefits (4 items), appearance (4 items), distress regarding family (3 items), and distress regarding recurrence (4 items). Higher scores represent a greater degree of the respective constructs. This questionnaire has demonstrated validity and reliability in cancer survivors [27], and in this study its internal consistency was high (α ≥ 0.80) for all subscales except for negative feelings (α = 0.74) and appearance (α = 0.56).

We administered the BSI to obtain a measure of psychological distress and disorder because previous literature has linked these challenges to endometrial and other gynecological cancer diagnoses [28–30], and because evidence indicates that physical activity may confer related benefits [31, 32]. The BSI is an 18-item measure in which participants are instructed to indicate how much they have been distressed by various factors over the previous seven days. Items are evenly split to comprise somatization (e.g., “Faintness or dizziness”), depression (e.g., “Feeling no interest in things”), and anxiety (e.g., “Nervousness or shakiness inside”) subscales. This measure has demonstrated validity and reliability in cancer survivor populations [33]; in this study its internal consistency was high for the depression (α = 0.85) and anxiety subscales (α = 0.83), but borderline for the somatization subscale (α = 0.62).

We included a measure of sleep quality and quantity in our analyses because this is an important aspect of quality of life for cancer survivors in the context of exercise interventions [34], and because physical activity has been linked to sleep quality [35]. The PSQI has respondents indicate how long it typically takes them to fall asleep and their typical sleep time via free-response questions, as well as additional items addressing various sleep issues. Higher scores indicate more problematic sleeping patterns. Previous literature has documented good reliability and validity of the PSQI in cancer survivors [36]; in this study its internal consistency was acceptable (α = 0.71).

Finally, we included a measure of perceived stress because it is an important aspect of psychological well-being [19] and because it has been linked to endometrial cancer survivorship [30]. The PSS is an instrument for measuring perception of stress that features 10 items to assess participants’ frequency of various stress-related feelings and thoughts over the last month. The PSS has demonstrated acceptable psychometric properties [37], and in this study its internal consistency was high (α = 0.85).

### Statistical methods

**Fig. 1:**
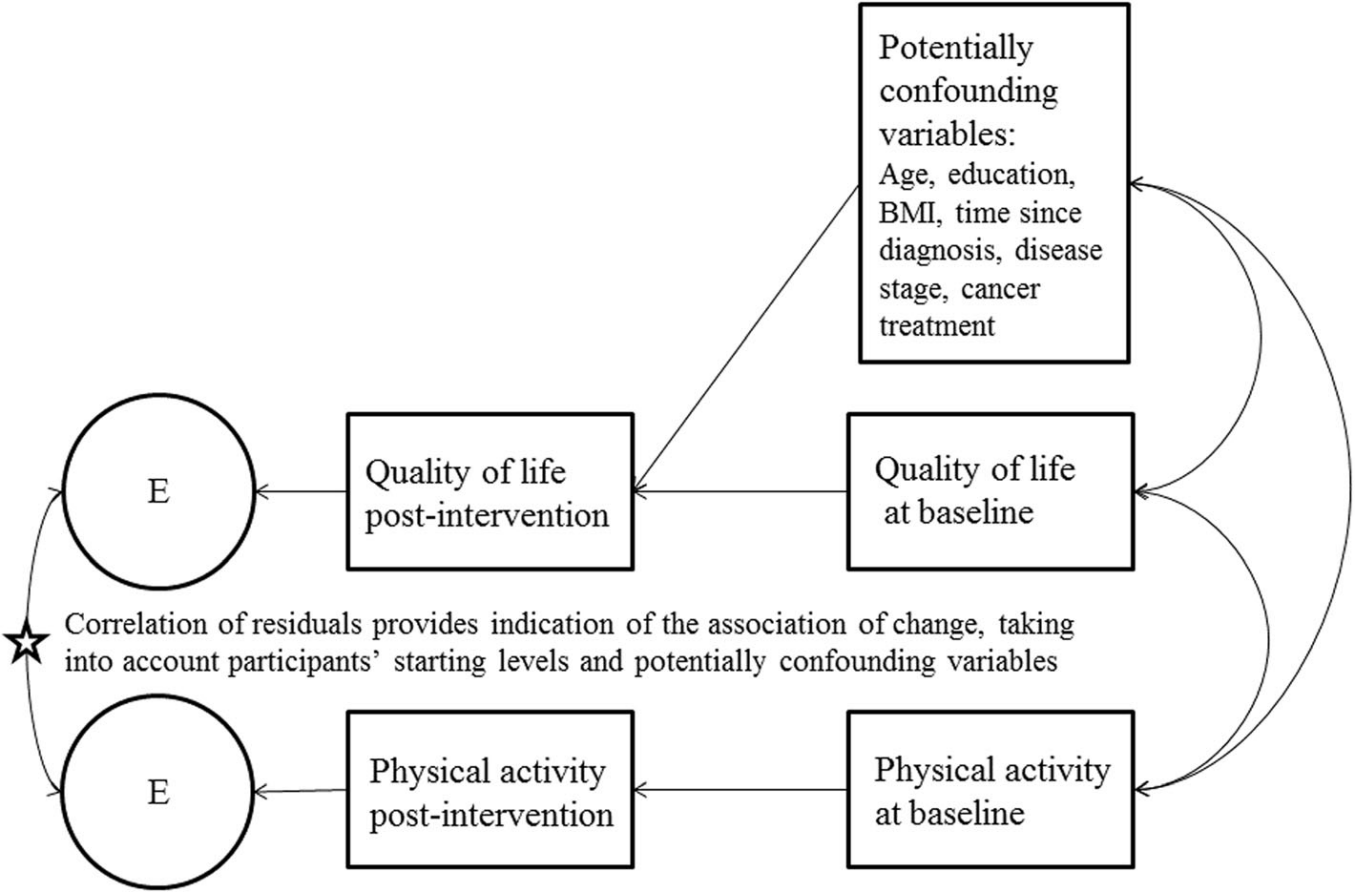
Model evaluating associations of change between physical activity and quality of life subscales

We took a residual score approach, conducting structural equation modeling path analyses to evaluate whether post-intervention physical activity was associated with post-intervention quality of life measures (Fig. 1) [38, 39]. All analyses took into account baseline levels of these factors and controlled for potentially confounding variables. We took this approach because taking into account baseline physical activity and quality of life levels is important in the context of behavioral interventions. Residual error terms for regression equations capture both random error and any persisting structure in the data that remains after regressing on independent variables. We evaluated the Pearson correlation of the residuals in order to provide information regarding the association between the *change* in the two constructs, taking into account participants’ starting points [38, 39]. In all analyses we controlled for age, education (high school or less vs. technical school or at least some college), body mass index (BMI; kg/m^2^), time since diagnosis, disease stage (1 vs 2 or 3), and cancer treatment (surgery only vs. surgery and other treatment). We selected these variables because, based on previous research, we expected them to be related to both physical activity and quality of life [40–44]. We also conducted post hoc analyses in which we added higher-order polynomial terms (quadratic and cubic) of physical activity to the models. To handle missing data, we assumed that data were missing at random [45] and used a random forest [46]-based imputation approach to predict missing values based on observed data [47]. This nonparametric approach is appropriate for imputing both continuous and categorical data and has been shown to compare favorably to other well-established methods of handling missing data [48, 49]. We set our nominal alpha level to 0.05 for all analyses, and adjusted for the inflated chance of type 1 error due to multiple testing using the Benjamini- Hochberg procedure [50]. All statistical analyses were performed using R version 3.3.2 [51].

## Results

**Fig. 2:**
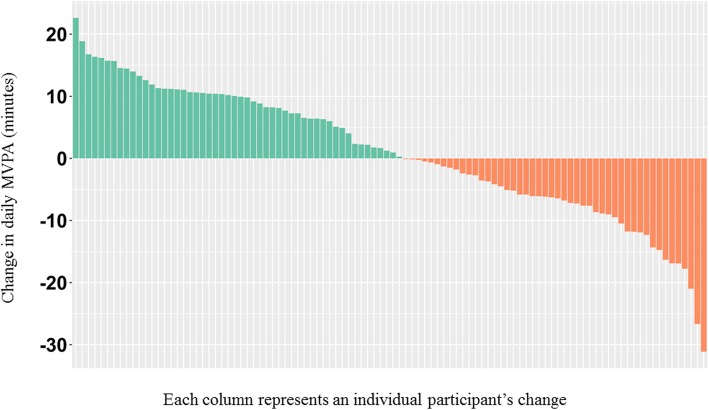
Participants’ change in moderate-to-vigorous physical activity (MVPA) ^a^Figure presenting imputed data (*n* = 100)

One hundred individuals completed baseline assessment and, of those, 74 completed the final assessment. Participants were mostly non-Hispanic white (80.0%) and well-educated (85.1% at least some college). The majority (75.7%) had stage I disease. The average time since diagnosis was 2.2 years. Participants were mostly overweight (26%) or obese (58%). At baseline, participants averaged 16.90 (SD = 10.43) minutes of moderate-to-vigorous physical activity (MVPA)/day, and at the follow-up assessment participants averaged 17.75 (SD = 9.65) minutes of MVPA/day. We plotted the difference of these scores to illustrate that about half of participants increased their physical activity over the course of the intervention (Fig. 2). Participants completed 61% of the scheduled phone counseling sessions in this study’s behavioral intervention.

After controlling for the false discovery rate, we identified associations between change in physical activity and change in four quality of life subscales (Table 1). Change in physical activity was positively (favorably) associated with change in SF-36 subscale scores for role limitations due to physical health and general health (*p* = .005 and *p* = .044, respectively). The correlation coefficient for change in physical activity and change in role limitations due to physical health was 0.29, while that for change in general health was 0.14. Furthermore, change in physical activity was negatively associated with change in pain as measured by QLACS (*p* = .041). The correlation coefficient for this relationship was − 0.22. Finally, change in physical activity was negatively associated with change in BSI scores reflecting somatic distress (*p* = .023). The correlation coefficient associated with this relationship was − 0.24. Change in physical activity was not statistically reliably associated with change in other aspects of quality of life after adjusting for the false discovery rate. Post hoc analyses did not provide evidence to indicate that adding higher order physical activity terms led to different conclusions.

**Table 1:** Residual covariance between post-intervention physical activity and quality of life, accounting for baseline levels^a^

Measure	Estimate^b^	SE	*P*-value	Adjusted P-value^c^
*SF-36*				
Physical Functioning	9.715	10.136	0.338	0.563
Mental Health	2.714	8.655	0.754	0.831
Vitality	19.732	11.188	0.078	0.243
Physical Role Limitation	85.154	23.035	<0.000	0.005
Bodily Pain	27.859	13.344	0.037	0.153
General Health	22.251	8.251	0.007	0.044
Social Functioning	9.093	12.273	0.459	0.648
Emotional Role Limitation	−15.454	21.230	0.467	0.648
*QLACS*				
Negative Feelings	−0.606	2.022	0.765	0.831
Positive Feelings	3.830	2.531	0.130	0.296
Cognitive Problems	−2.887	2.006	0.150	0.313
Pain	−7.234	2.575	0.005	0.041
Sexual Interest	2.200	2.700	0.415	0.648
Energy Fatigue	−3.406	1.926	0.077	0.243
Social Avoidance	−5.368	2.170	0.013	0.067
Financial Problems	1.242	2.214	0.575	0.756
Benefits	0.124	3.049	0.967	0.969
Distress Family	−4.605	3.034	0.129	0.296
Appearance	0.059	1.528	0.969	0.969
Distress Recurrence	−4.042	2.625	0.124	0.296
*BSI*				
Somatization	−4.457	1.433	0.002	0.023
Depression	−2.374	1.892	0.209	0.374
Anxiety	−2.370	1.749	0.175	0.337
*PSS*				
Perceived Stress Score	−1.423	3.776	0.706	0.831
*PQSI*				
Global Score	−0.693	2.096	0.741	0.831

^a^All models adjusted for age, education, BMI, time since diagnosis, disease stage, and treatment

^b^Residual covariance estimates obtained via path analyses for post-intervention physical activity and post-intervention quality of life measures, taking in to account baseline levels and potentially confounding variables; results are for imputed data

^c^P-values were adjusted for false discovery rate using the Benjamini and Hochberg procedure [50]

## Discussion

In our sample of endometrial cancer survivors receiving a physical activity intervention, increasing physical activity was positively associated with improvements in role limitation due to physical health, general health, pain, and somatic distress. These associations between physical activity and physical health are consistent with previous literature among survivors of gynecological cancers [52] and other types of cancer [53]. We did not find evidence to indicate that change in physical activity was associated with emotional or mental health indicators after controlling for the false discovery rate. Taken together, our findings are consistent with cross-sectional studies [54] and may provide some insight as to why previous randomized control trials with endometrial cancer survivors have produced mixed results regarding the impact of physical activity intervention on global quality of life [11, 12, 55]. It may be that physical activity change in this population is apt to lead to physical benefits more readily than mental and emotional health benefits.

Analyses of the parent study published previously indicate that the time trend for physical activity was quadratic; increases in the adoption of physical activity were observed that were not maintained by all participants at the six month follow-up assessment. Unfortunately, this is common in physical activity interventions. The observed variation in physical activity is perhaps a strength of the present study as it allows for a better sense as to how quality of life constructs may change along with both a net increase and a net decrease in physical activity over six months. While there is a substantial body of evidence indicating that physical activity can confer mental and emotional benefits in the general population [56], it is not clear what dose of physical activity is needed to produce changes in different aspects of quality of life, and how physical activity dosage might differentially affect aspects of quality of life in endometrial cancer survivors. Participants in the current study were active (moderate-to-vigorous intensity) for an average of approximately 125 min per week at the end of the six-month intervention period. This is below the recommended level of 150 min per week [57]. The intervention target of this study was to increase moderate-intensity walking, but it is possible that a higher volume of physical activity is required to yield beneficial changes. Results from a study conducted by Thraen-Borowski and colleagues (2013) suggested the presence of a dose-response relationship between physical activity and physical quality of life in cancer survivors, although this relationship is less clear for quality of life related to social and mental health constructs [58]. To explore what role physical activity dosage may play in affecting quality of life outcomes in this population, we evaluated all models with high order physical activity terms (quadratic and cubic); results did not lead to changes in study conclusions. Nonetheless, it may be reasonable to expect that quality of life benefits in this population are accrued in a dose-dependent fashion as they are in individuals without a history of cancer diagnosis [59], and intervention efforts should be centered on gradually increasing physical activity to at least nationally recommended levels. It is possible that different types and contexts of physical activity might uniquely impact various aspects of quality of life in this population. For example, more social forms of physical activity (e.g., group/partnered walking) appear to have a stronger impact on mental health-related aspects of quality of life than standard activity in colorectal cancer survivors [58], and more explicitly mindful physical activity (e.g., yoga) may be particularly beneficial for improving sleep and decreasing distress in cancer survivors [60].

In the current study we observed two relationships between change in physical activity and change in specific aspects of quality of life that were statistically significant before adjusting for multiple testing, but not after. These were for favorable relationships between change in physical activity and SF-36 subscale-measured bodily pain and QLACS-measured social avoidance (Table 1). The former finding would corroborate the current study’s finding regarding QLACS-measured pain, and the conclusion that change in physical activity may be negatively associated with pain in this population. The notion that physical activity is negatively associated with pain is supported by meta-analysis of quantitative studies [34] and a meta-synthesis of qualitative data in cancer survivors generally [61], but less research has investigated this question in endometrial cancer survivors specifically. The apparent discrepancy in the current study between the SF-36 subscale measure of bodily pain and the QLACS measure of pain may be due to the fact that the former is a subtly different construct from the latter. The SF-36 asks more generally “*How much bodily pain have you had during the past 4 weeks?*”, whereas the QLACS asks participants to indicate to what degree physical pain *interferes* with quality of life (e.g., *To what degree is it true for you that: You were bothered by pain that kept you from doing the things you wanted to do*.) While the two findings are trending towards concordance, it may be that physical activity is more closely associated with benefits regarding effective pain management than with its cessation. The trend regarding QLACS-measured social avoidance highlights the possibility that additional social quality of life-related benefits of physical activity may exist for endometrial cancer survivors, and invites further research.

A limitation of this study is that data were collected as part of a single-arm intervention trial, so changes over time cannot be compared to a control group. The study design precludes establishing temporal precedence; some evidence suggests that a reduction of pain could precipitate a subsequent change in physical activity [62]. Still, it may be reasonable to expect that physical activity plays a causal role in affecting quality of life in endometrial cancer survivors, as convincing evidence suggests that it does in cancer survivors generally [34]. Our study is limited in terms of generalizability due to the fact that our sample was largely non-Hispanic white and particularly well- educated. Another important issue to consider is the heterogeneity in cancer stage at diagnosis and cancer treatment. We adjusted all analyses for these factors, but the threat of residual confounding remains, and there is a possibility that the effect of physical activity on endometrial cancer survivors’ quality of life is modified by these and other factors. The sample size of the current study precluded the investigation of these research questions, but future research should consider stratifying analyses by these variables. The reader is referred to McAlpine and colleagues (2014) to guide these and other methodological considerations in the context of quality of life-related research in endometrial cancer survivors [63]. Findings of this study need to be interpreted in light of the potential for selection bias. Participants, who may have been particularly motivated to participate in a physical activity-related intervention, may have been systematically different from the average endometrial cancer survivor. Furthermore, a relatively high percentage (26%) of participants was lost due to follow up. The most common reason given for this was a lack of time, and there were also some withdrawals due to medical reasons. Though we used advanced imputation methods to handle missing data, doing so still requires making unverifiable assumptions. Also, given that the parent study was an exercise intervention, it is possible that this relatively well-educated sample could have been influenced by response bias if they anticipated the research questions when reporting their quality of life end points. For example, if they were knowledgeable of the benefits of physical activity and felt they had not increased their activity levels, they might have been more likely to indicate lower quality of life scores. To minimize this threat, we used measures with established psychometric properties; the internal consistency of most subscales in this sample was high, but in some cases it was less than ideal (see Methods section). Studies with stronger experimental design features are needed to advance this line of research.

## Conclusions

Increased physical activity was associated with a favorable change in role limitations due to physical health, general health, and somatic distress. We did not find evidence to suggest that physical activity is statistically reliably associated with emotional or mental health indicators of quality of life. Additional research is needed to better characterize the relationships between change in various aspects of quality of life and specific types and doses of physical activity in endometrial cancer survivors.

## Abbreviations

BMI: Body mass index
BSI: Brief Symptom Inventory-18
EMA: Electronic momentary assessment
MVPA: Moderate-to-vigorous physical activity
PSQI: Pittsburgh sleep quality index
PSS: Perceived stress scale
QLACS: Quality of Life in Adult Cancer Survivors questionnaire
SF-36: 36-Item short form survey
